# Binding Mechanism and Taste-Masking Effect of Milk Proteins with Flavonoids from Pandan Revealed by Spectroscopic and Electronic Tongue Analysis

**DOI:** 10.3390/foods15162870

**Published:** 2026-08-17

**Authors:** Junyi Zhang, Xiaowei Qin, Zhen Feng, Shuzhen He, Guanhua Lou, Wei Cheng, Fei Liu, Chunhe Gu

**Affiliations:** 1 Key Laboratory of Dairy Science, Ministry of Education, College of Food Science, Northeast Agricultural University, 600 Changjiang Road, Harbin 150030, China; 2 Spice and Beverage Research Institute, Chinese Academy of Tropical Agricultural Sciences, Wanning 571533, China

**Keywords:** β-lactoglobulin, β-casein, flavonoids, molecular docking, taste masking, electronic tongue

## Abstract

With the growing interest in pandan-based products, bitterness and astringency associated with flavonoids may limit their sensory acceptance. This study investigated the interactions and taste-modulating effects of two milk proteins, β-casein (β-CN) and β-lactoglobulin (β-LG), with two representative bitter flavonoids, catechin (C) and naringin (NAR), in aqueous model systems. Fluorescence spectroscopy showed that both flavonoids produced concentration-dependent quenching of the milk proteins. β-CN exhibited more pronounced interaction-related spectroscopic responses than β-LG, which may be associated with its flexible and intrinsically disordered structure. Molecular docking predicted hydrogen-bonding and hydrophobic interactions in all four protein–flavonoid systems, with catechin and naringin interacting mainly with the internal hydrophobic cavity of β-LG and surface-exposed regions of the β-CN model. Circular dichroism and Fourier-transform infrared spectroscopy indicated ligand-dependent structural changes. For β-LG, catechin slightly decreased the estimated antiparallel and total β-sheet fractions, whereas naringin produced a modest increase. For β-CN, catechin produced a more apparent redistribution between the estimated α-helix and β-sheet fractions, while naringin caused comparatively smaller changes. Electronic tongue measurements showed that the addition of the milk proteins reduced the bitterness- and astringency-related sensor responses of catechin and naringin. Under the tested conditions, β-CN produced greater attenuation of these responses than β-LG, while the umami-related response remained comparatively high. These findings support the potential application of milk proteins as taste-modulating components in flavonoid-containing dairy formulations, although validation in real food matrices is required.

## 1. Introduction

*Pandanus amaryllifolius* Roxb., commonly known as pandan, is a perennial tropical plant belonging to the family Pandanaceae. Its leaves are widely used as a natural food flavoring in tropical and subtropical regions because they impart a characteristic aroma resembling that of fragrant rice [1]. Pandan leaves contain various bioactive constituents, including alkaloids, polyphenols, and flavonoids [2]. Previous HPLC profiling of pandan leaves collected from three locations in Malaysia identified catechin and naringin among the quantified flavonoids. Catechin concentrations ranged from 0.153 to 0.613 mg/g dry weight, whereas naringin concentrations of 0.223–0.325 mg/g dry weight were detected in samples from Bachok and Klang but not in the sample from Pontian, indicating geographical variation in flavonoid composition [3,4,5]. Catechin and naringin were therefore selected as compositionally relevant model flavonoids previously reported in pandan rather than as compounds representing its complete flavonoid profile.

The two compounds also provide contrasting structural and sensory models. Catechin is a relatively small, non-glycosylated flavan-3-ol containing multiple hydroxyl groups and is associated with bitterness and astringency. In contrast, naringin is a larger flavanone glycoside containing a bulky sugar moiety and is characterized by pronounced bitterness [3,4,5]. These differences in molecular size, glycosylation, and hydroxyl-group distribution make them suitable models for investigating how flavonoid structure influences interactions with food proteins and the corresponding taste-related sensor responses.

Flavonoids are plant secondary metabolites characterized by a C6–C3–C6 carbon skeleton and have attracted considerable interest in functional foods and nutraceutical products [6,7]. However, their application in food systems may be limited by physicochemical instability, restricted bioavailability, and undesirable sensory attributes, particularly bitterness and astringency [8]. Interactions with dietary proteins may provide a strategy for modulating these properties. Through hydrogen bonding, hydrophobic interactions, and other non-covalent forces [9,10], proteins may alter the molecular environment and accessibility of flavonoids, potentially reducing their availability for interactions associated with bitter and astringent responses [11,12].

The incorporation of pandan-associated bioactive compounds into dairy formulations creates opportunities for interactions between flavonoids and milk proteins. Milk proteins consist primarily of caseins and whey proteins, accounting for approximately 80% and 20% of the total protein fraction, respectively [13]. β-Casein (β-CN) is an intrinsically disordered protein with high conformational flexibility and exposed hydrophobic regions, facilitating its association with structurally diverse ligands [13,14]. By contrast, β-lactoglobulin (β-LG), the major whey protein, is a compact globular protein belonging to the lipocalin family and contains an internal hydrophobic cavity capable of accommodating various hydrophobic compounds, including polyphenols, fatty acids, and vitamins [14,15].

Although previous studies have extensively characterized milk protein–polyphenol interactions, most have focused on predicted or experimentally measured binding parameters and protein structural changes [14,16,17,18]. Comparatively less attention has been given to whether the contrasting structural characteristics of β-CN and β-LG are associated with differences in flavonoid taste modulation. β-CN and β-LG therefore provide two distinct protein models for examining how protein organization may influence interaction-related structural responses and bitterness- and astringency-related sensor signals [19].

Accordingly, this study investigated the interactions of β-CN and β-LG with catechin and naringin in aqueous model systems and evaluated their effects on electronic-tongue responses. Fluorescence spectroscopy, UV–Vis spectroscopy, circular dichroism, Fourier-transform infrared spectroscopy, particle characterization, molecular docking, and electronic tongue analysis were combined to examine protein-dependent interaction patterns, conformational and colloidal changes, and their exploratory associations with taste-related sensor responses. This study does not aim to reproduce the complete composition of pandan or directly measure human taste perception; rather, it uses two flavonoids previously reported in pandan as structurally and sensory-distinct model compounds. The findings may support the rational use of milk proteins as taste-modulating components in flavonoid-containing dairy formulations.

## 2. Materials and Methods

### 2.1. Chemical Reagents

Naringin (NAR; ≥98%), catechin (C; ≥98%), β-casein (β-CN; ≥98%), and β-lactoglobulin (β-LG; ≥90%) were purchased from Shanghai Yuanye Bio-Technology Co., Ltd. (Shanghai, China). All other chemicals and reagents were of analytical grade. and all other reagents used in this study were purchased from Shanghai Yuanye Bio-Technology Co., Ltd. (Shanghai, China).

### 2.2. Sample Preparation

Protein solutions were prepared by dissolving the proteins in 10 mL of 10 mM phosphate-buffered saline (PBS, pH 7.0) and stored at 4 °C for subsequent use. The final protein concentration was adjusted to 50 μmol/L for the preparation of protein–flavonoid interaction samples. PBS (10 mM, pH 7.0) was used as the blank medium for all interaction experiments.

Flavonoid stock solutions (10 mmol/L) were prepared by dissolving the flavonoids in dimethyl sulfoxide (DMSO) and stored at 4 °C. Prior to the experiments, the stock solutions were diluted with phosphate-buffered saline (PBS) to achieve final flavonoid concentrations of 0, 50, 100, 250, and 500 μmol/L in the reaction mixtures. The final DMSO concentration was strictly maintained at ≤5% (*v*/*v*) across all samples to prevent protein denaturation. Subsequently, the diluted flavonoid solutions were mixed with the protein solutions at a 1:1 (*v*/*v*) ratio and incubated for a minimum of 30 min with gentle agitation to reach binding equilibrium prior to further measurements.

### 2.3. UV-Vis Absorption Spectroscopy

The absorbance was measured using an ultraviolet–visible spectrophotometer (UV-2700, Shimadzu Instruments (Suzhou) Co., Ltd., Suzhou, China). The flavonoids, casein, and whey protein were dissolved in 0.1 mol/L phosphate-buffered saline (PBS) containing 5% (*v*/*v*) dimethyl sulfoxide (DMSO). UV-Vis absorption spectra of the samples were recorded at room temperature using a UV-Vis-NIR spectrophotometer. A 0.1 mol/L PBS solution containing 5% (*v*/*v*) DMSO served as the blank reference. All spectra were collected over a wavelength range of 250–320 nm.

### 2.4. Fourier Transform Infrared Spectroscopy (FT-IR)

Fourier transform infrared (FTIR) spectroscopy measurements were performed using a Nicolet 6700 FTIR spectrometer (Thermo Fisher Scientific, Waltham, MA, USA). Spectra were recorded in the wavenumber range of 4000–400 cm^−1^ with a resolution of 4 cm^−1^. For each sample, 20 scans were collected and averaged to improve the signal-to-noise ratio. A background spectrum of ambient air was recorded prior to sample measurement to eliminate environmental interference. Functional group assignments were made by comparing the observed peak wavenumbers with established literature values. All spectra were processed and visualized using Spectragryph software (version 1.2.16.1).

### 2.5. Circular Dichroism Spectroscopy (CD)

Far-UV circular dichroism (CD) spectra of the samples were recorded using a Jasco J-1700 spectropolarimeter (Jasco, Tokyo, Japan). Measurements were conducted over a wavelength range of 190–260 nm at a scanning speed of 100 nm/min. The relative fractions of protein secondary structures were determined using the instrument’s built-in secondary structure estimation software, based on the Yang reference algorithm. The protein secondary structure contents calculated by spectral deconvolution using CDNN software (version 2.1.0.223; Gerald Böhm, Martin Luther University Halle-Wittenberg, Halle, Germany) are presented in Table 1.

### 2.6. Intrinsic Fluorescence Spectroscopy

Changes in the intrinsic fluorescence of β-casein (β-CN) and β-lactoglobulin (β-LG) following the addition of catechin or naringin were measured using an F-7000 fluorescence spectrophotometer (Hitachi, Tokyo, Japan). The protein solutions were separately diluted to a final concentration of 1 μmol/L in phosphate buffer (PB). Catechin and naringin stock solutions were independently titrated into the protein solutions to obtain ligand-to-protein molar ratios of 0:1, 1:1, 2:1, 5:1, and 10:1. The same preparation procedure and final experimental conditions were used for both flavonoids. For each ligand concentration, corresponding PB solutions containing catechin or naringin without protein were prepared as ligand blanks.

The excitation wavelength was set to 295 nm, and the emission spectra were recorded from 310 to 500 nm at room temperature. The scan rate was 1200 nm/min, both excitation and emission slit widths were 5 nm, and the photomultiplier tube voltage was 700 V. The spectrum of the corresponding ligand blank was subtracted from each protein–ligand spectrum. The resulting fluorescence intensities were subsequently corrected for the inner filter effect using the corresponding UV–Vis absorbance data. All measurements were performed in triplicate.

### 2.7. Particle Size and Zeta Potential

The colloidal characteristics of the aqueous protein–flavonoid systems were evaluated by measuring their particle size distributions and zeta potentials at 25 °C. Zeta potential measurements were performed using a Zetasizer Pro (Malvern Panalytical, Malvern, UK). Particle size distributions were determined using a static multi-angle light-scattering particle size analyzer (Malvern Panalytical, Malvern, UK).

To minimize multiple-scattering effects, the β-CN, β-LG, and corresponding protein–polyphenol samples were diluted 1000-fold (1:1000, *v*/*v*) with ultrapure water before measurement. The refractive indices of the dispersed protein-containing phase and the aqueous dispersant were set to 1.46 and 1.33, respectively. Particle size was expressed as modal particle diameter, and zeta potential was reported in mV. Each sample was measured 3 times, and the results are presented as mean ± standard deviation.

### 2.8. Molecular Docking

Molecular docking was performed to investigate the potential binding modes and interactions of catechin and naringin with β-casein (β-CN) and β-lactoglobulin (β-LG). The three-dimensional structures of catechin and naringin were obtained from the PubChem database. The X-ray crystal structure of bovine β-LG was obtained from the RCSB Protein Data Bank (PDB ID: 1BSY). The predicted three-dimensional structure of bovine β-CN was obtained from the AlphaFold Protein Structure Database using the UniProt accession P02666. Because β-CN is intrinsically disordered, the predicted structure was used as a computational receptor model rather than an experimentally determined native structure.

Before docking, the protein structures were prepared by removing water molecules and unnecessary small molecules, adding hydrogen atoms, and performing structural optimization. The ligand structures were converted into docking-compatible formats after energy minimization.

Docking simulations were carried out using AutoDock Vina 1.2.7. The docking search region was defined around the potential ligand-binding regions of the proteins, and multiple conformations were generated for each protein–flavonoid complex. The docking pose with the lowest binding energy and reasonable interaction pattern was selected as the representative binding model.

The binding affinity was evaluated based on the binding energy calculated by AutoDock Vina and expressed as kcal/mol. More negative binding energy values indicate stronger predicted binding affinity between flavonoids and proteins. The interactions between flavonoids and proteins, including hydrogen bonds, hydrophobic interactions, and other non-covalent interactions, were visualized and analyzed using Discovery Studio Visualizer 2019 (Dassault Systèmes BIOVIA, San Diego, CA, USA).

### 2.9. Electronic Tongue Analysis

The electronic-tongue measurements were performed using β-CN–C2, β-LG–C2, β-CN–NAR2, and β-LG–NAR2. These formulations were prepared at a protein-to-flavonoid molar ratio of 1:2, equivalent to a flavonoid-to-protein molar ratio of 2:1. The suffixes C2 and NAR2 therefore indicate two moles of catechin or naringin per mole of protein. Free catechin (C) and naringin (NAR) solutions containing the same final flavonoid concentrations as the corresponding protein-containing samples were analyzed as controls.

The taste-related responses were evaluated using an SA402B taste-sensing system (Insent, Atsugi, Japan), with particular attention to bitterness, astringency, umami, and aftertaste-related responses. Before measurement, the sensors were activated and calibrated in the reference solution until the membrane-potential fluctuation was below 0.5 mV.

The sensors were first equilibrated in a fresh reference solution to record the initial reference potential (Vr) and were then immersed in the sample solution to obtain the sample potential (Vs). The initial taste response was calculated as Vs − Vr. After sample measurement, the sensors were rinsed and transferred to a fresh reference solution to determine the new reference potential (Vr′). The change in membrane potential caused by adsorption (CPA), representing the aftertaste-related response, was calculated as Vr′ − Vr. The first measurement cycle was discarded to minimize sensor-adaptation effects, and the final two cycles were averaged. The change in membrane potential caused by adsorption (CPA), representing the persistence of adsorbed taste substances on the sensor membrane, was calculated as Vr′ − Vr. CPA values obtained using the C00 and AE1 sensors were used to characterize bitterness- and astringency-related aftertaste responses, respectively. All samples were analyzed in triplicate.

### 2.10. Integrated Structure–Electronic-Tongue Analysis

Electronic-tongue data and corresponding structural measurements were directly matched for four protein–flavonoid formulations: β-CN–C2, β-LG–C2, β-CN–NAR2, and β-LG–NAR2. Changes in total β-sheet content, relative fluorescence intensity, particle size (ΔlnD), and zeta potential (Δζ) were calculated relative to the corresponding protein controls. Apparent reductions in bitterness and astringency and the change in umami response were calculated relative to the corresponding free-flavonoid controls.

Because only four matched formulations were available, the sample size was considered insufficient for reliable inferential correlation analysis. Therefore, an exploratory descriptive integration was performed. Each variable was standardized across the four formulations using Z-score transformation, and the resulting standardized profiles were visualized as an integrated heatmap. No statistical significance or causal relationship was assigned to the observed patterns.

### 2.11. Statistical Analysis

Except for the data presented in Table 2, all experimental measurements were performed using three independently prepared samples (*n* = 3), and the results are expressed as mean ± standard deviation (SD). Mean values and SDs were calculated using Microsoft Excel (Microsoft Corporation, Redmond, WA, USA). Statistical analysis was applied only to data obtained from independent replicates; the data in Table 2 were reported descriptively and were not subjected to inferential statistical analysis. One-way ANOVA followed by Duncan’s multiple range test was performed using SPSS Statistics version 25.0 (IBM Corp., Armonk, NY, USA). Differences were considered significant at *p* < 0.05. All figures were generated using Origin 2021 (OriginLab Corp., Northampton, MA, USA).

## 3. Results and Discussion

### 3.1. UV-Vis Absorption Spectroscopy Analysis

UV–Vis spectra were evaluated over the experimentally displayed wavelength range of 245–330 nm. The absorption of proteins near 280 nm is mainly associated with the π → π* transitions of aromatic amino acid residues, particularly Trp and Tyr [20]. However, catechin and naringin also absorb within this wavelength region. Because the corresponding flavonoid-only spectra were not subtracted from the protein–flavonoid spectra, the measured curves represent the combined absorbance of the protein and flavonoid components. The reported maximum absorption wavelengths (λmax) and maximum absorbance values (Amax) were therefore treated as apparent values of the mixtures and interpreted descriptively [20].

**Figure 1:**
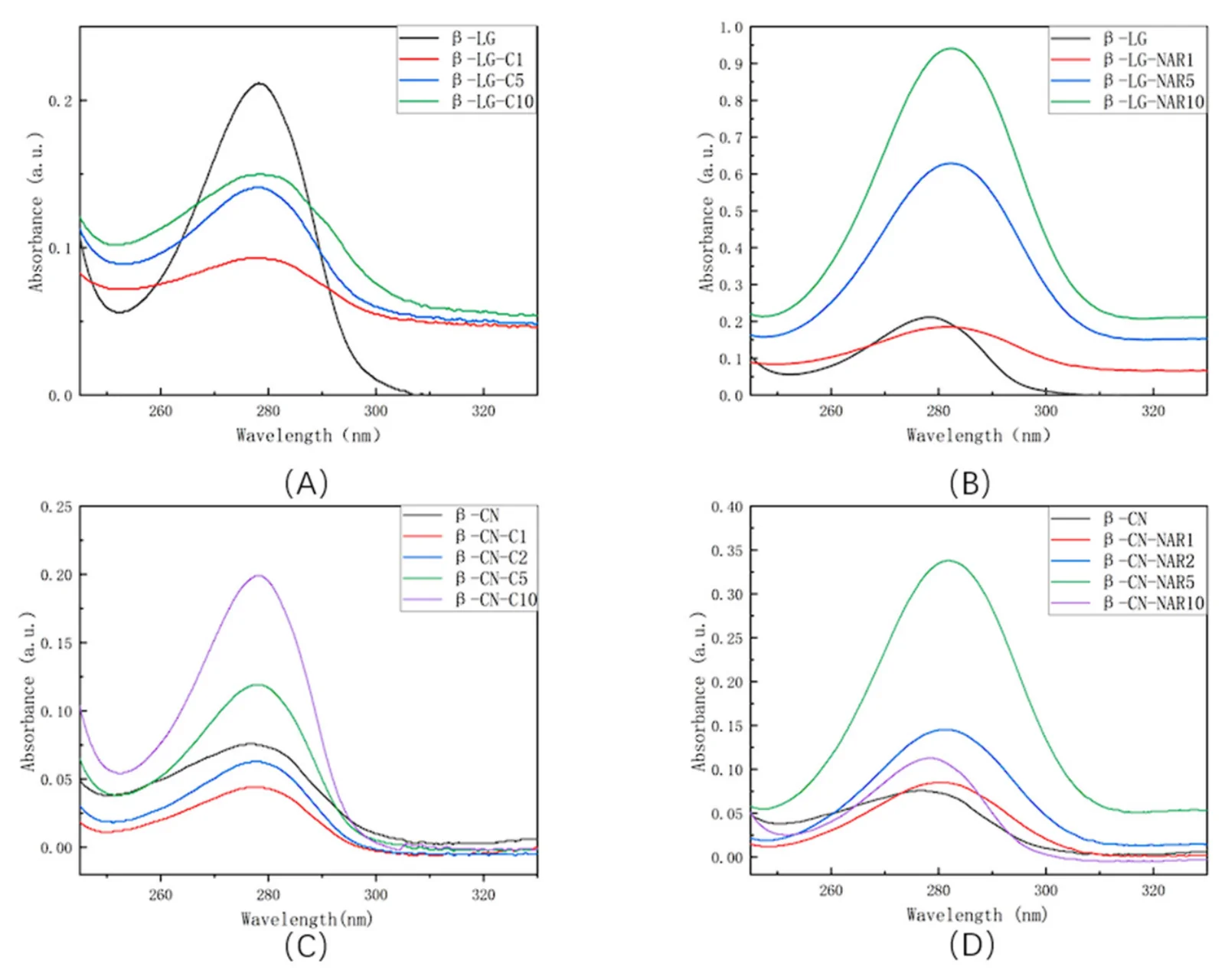
UV–Vis absorption spectra of β-lactoglobulin (β-LG) and β-casein (β-CN) in the absence and presence of catechin (C) or naringin (NAR) at different polyphenol-to-protein molar ratios: (**A**) β-LG–catechin; (**B**) β-LG–naringin; (**C**) β-CN–catechin; and (**D**) β-CN–naringin. The complete spectra were recorded from 200 to 330 nm, while the region from 245 to 330 nm is displayed for clarity. The numerical suffixes indicate the corresponding polyphenol-to-protein molar ratios.

Native β-LG exhibited an apparent λmax of 278.3 nm and an Amax of 0.212. The apparent λmax values of β-LG–C1, β-LG–C5, and β-LG–C10 were 277.7, 278.1, and 278.5 nm, respectively, with corresponding Amax values of 0.093, 0.141, and 0.150 (Figure 1A). These values represented only small and non-directional changes in the apparent peak position. For β-LG–NAR1, β-LG–NAR5, and β-LG–NAR10, the apparent λmax values were 281.7, 282.3, and 282.4 nm, respectively, with corresponding Amax values of 0.185, 0.629, and 0.941 (Figure 1B). The marked increase in absorbance at the higher naringin ratios likely included an increasing contribution from naringin itself; therefore, it was not interpreted as a quantitative change in protein absorbance.

Native β-CN exhibited an apparent λmax of 276.8 nm and an Amax of 0.076. Following catechin addition, the apparent λmax values of β-CN–C1, β-CN–C2, β-CN–C5, and β-CN–C10 were 277.5, 277.8, 278.0, and 278.2 nm, respectively, with corresponding Amax values of 0.044, 0.063, 0.119, and 0.199 (Figure 1C). For β-CN–NAR1, β-CN–NAR2, β-CN–NAR5, and β-CN–NAR10, the apparent λmax values were 280.5, 281.3, 281.9, and 278.5 nm, respectively, with corresponding Amax values of 0.085, 0.145, 0.338, and 0.113 (Figure 1D). The non-monotonic changes in both peak position and absorbance indicate that the spectral responses were influenced by the composition of the mixtures.

Overall, the UV–Vis spectra showed composition-dependent changes in the apparent absorption profiles of the protein–flavonoid mixtures. Because of spectral overlap and the absence of matched flavonoid-background subtraction, these changes do not independently demonstrate protein conformational alterations or specific binding interactions. The UV–Vis results were therefore considered only as complementary observations together with the fluorescence, CD, and molecular docking results.

### 3.2. Fourier Transform Infrared Spectroscopy (FT-IR) Analysis

**Figure 2:**
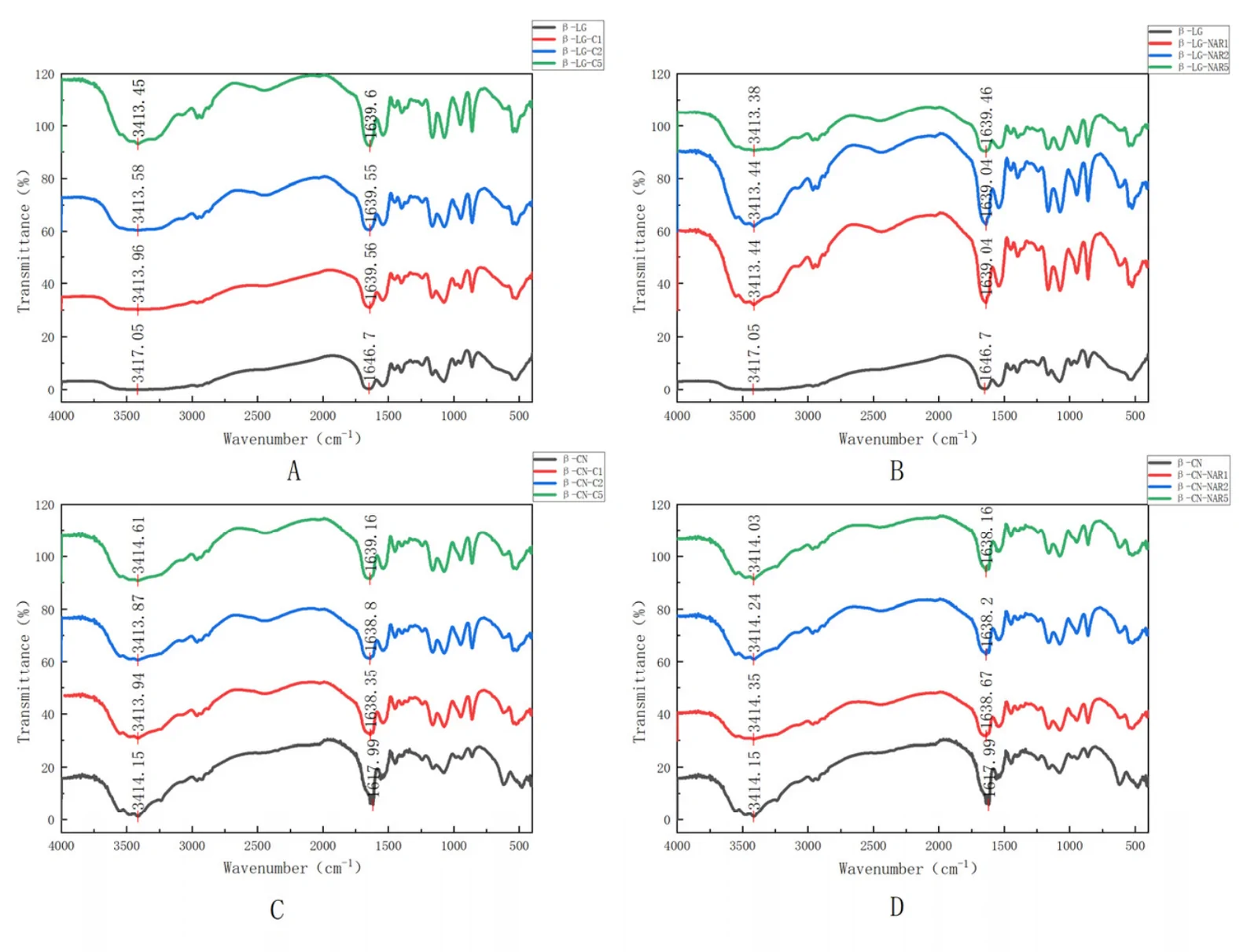
Fourier-transform infrared (FTIR) spectra of milk proteins in the absence and presence of flavonoids. (**A**) β-LG–C, (**B**) β-LG–NAR, (**C**) β-CN–C, and (**D**) β-CN–NAR; β-CN, β-casein; β-LG, β-lactoglobulin; C, catechin; NAR, naringin.

Fourier transform infrared (FTIR) spectroscopy was employed to investigate the structural changes and intermolecular interactions of milk proteins following polyphenol binding. The amide I band (1600–1700 cm^−1^), primarily attributed to C=O stretching vibrations of the peptide backbone, is highly sensitive to changes in protein secondary structure [13]. FTIR spectra were collected over the range of 4000–1000 cm^−1^. As shown in Figure 2, the characteristic amide I band of the native proteins appeared at approximately 1638 cm^−1^, and changes in its position and intensity after complex formation reflected alterations in the protein conformation induced by catechin and naringin [21].

As shown in Figure 2, the FTIR spectra of the native proteins exhibited characteristic absorption bands at approximately 1635–1650 cm^−1^ (amide I), primarily attributed to C=O stretching vibrations of the peptide backbone, and 1530–1545 cm^−1^ (amide II), arising mainly from N–H bending coupled with C–N stretching vibrations. In addition, a broad absorption band centered at 3300–3450 cm^−1^ was assigned to the stretching vibrations of O–H and N–H groups, which are associated with the hydrogen-bonding network within the protein matrix [22].

The addition of flavonoids resulted in noticeable changes in the characteristic FTIR bands, suggesting the formation of protein–polyphenol complexes through non-covalent interactions.

For the β-casein–catechin system, the O–H/N–H stretching band shifted from 3411.9 to 3286.7 cm^−1^ with increasing catechin concentration. This pronounced red shift is consistent with enhanced hydrogen-bonding interactions between the phenolic hydroxyl groups of catechin and the polar amino acid residues of β-casein. As the catechin-to-protein molar ratio increased to 5:1, the amide I and amide II bands gradually decreased in intensity and became broader, indicating changes in the local environment of the peptide backbone and conformational rearrangements associated with protein–catechin complex formation. Moreover, an elevated spectral baseline was observed at higher catechin concentrations, which may be attributed to the formation of larger protein–polyphenol aggregates, leading to increased light scattering during FTIR measurements.

In the amide I region, the β-CN–catechin system exhibited a noticeable shift from 1635.9 to 1643.7 cm^−1^. This blue shift suggests alterations in the local environment of the peptide backbone and is indicative of conformational rearrangements within the protein. Together with the CD results, these spectral changes may reflect an increase in structural disorder, accompanied by a higher proportion of random coil structures. In contrast, the amide I band of the β-LG–naringin system shifted slightly toward lower wavenumbers, from 1637.7 to 1635.7 cm^−1^. Combined with the CD results, this finding suggests that naringin induces only subtle local conformational rearrangements in β-lactoglobulin while preserving its overall secondary structure [17].

### 3.3. Circular Dichroism Spectroscopy (CD) Analysis

**Figure 3:**
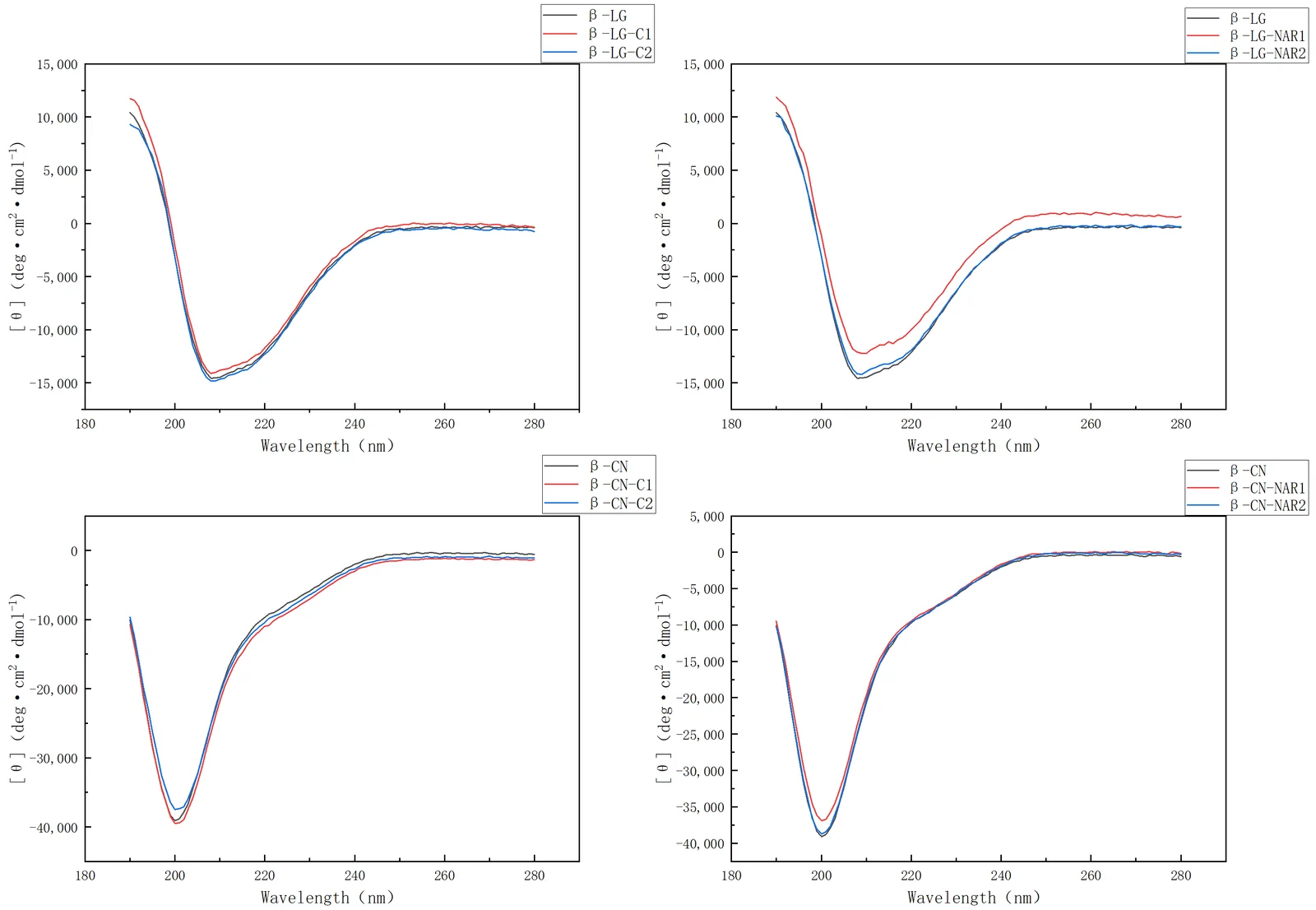
Far-ultraviolet circular dichroism (CD) spectra of milk proteins in the absence and presence of flavonoids. β-LG–C, β-LG–NAR, β-CN–C, and β-CN–NAR; β-casein; β-LG, β-lactoglobulin; C, catechin; NAR, naringin.

Far-ultraviolet circular dichroism (far-UV CD) spectroscopy (190–260 nm) was employed to evaluate changes in the secondary structures of milk proteins following polyphenol binding [23]. As shown in Figure 3, β-casein (β-CN) exhibited a pronounced negative ellipticity centered at approximately 200 nm, which is characteristic of intrinsically disordered proteins. This spectral feature indicates that β-CN predominantly adopts a random coil conformation under the experimental conditions.

In contrast, β-lactoglobulin (β-LG) exhibited a distinct negative ellipticity near 210 nm and a positive band at 190–195 nm, consistent with its β-sheet-rich globular structure. The secondary structure analysis further confirmed that β-sheet was the predominant structural element, accompanied by smaller proportions of α-helices and β-turns.

The addition of catechin (C) and naringin (NAR) resulted in distinct changes in the CD spectra of β-CN, suggesting different modes of protein–polyphenol interaction. In the β-CN–catechin system, a concentration-dependent structural response was observed. At a low catechin-to-protein molar ratio (β-CN–catechin 1), the negative ellipticity at approximately 200 nm increased slightly, indicating a subtle change in the conformational flexibility of β-CN. As the catechin concentration increased (β-CN–catechin 2), the negative ellipticity decreased markedly, suggesting reduced structural disorder and the formation of a more ordered protein conformation. A similar trend was observed for the β-CN–naringin system, in which the decreased intensity of the random coil signal indicates that naringin promotes structural stabilization of β-CN through non-covalent intermolecular interactions [24]. Both the β-LG–catechin and β-LG–naringin systems exhibited a decrease in negative ellipticity near 210 nm, indicating subtle alterations in the secondary structure of β-lactoglobulin following polyphenol binding. In addition, a slight red shift in the characteristic band was observed, suggesting local conformational rearrangements within the protein. These spectral changes, together with the FTIR results, are consistent with polyphenol-induced modifications of the hydrogen-bonding environment and non-covalent interactions that contribute to structural stabilization of the protein–polyphenol complexes [23].

Secondary structure contents were estimated using CDNN 2.1.0.223 software through spectral deconvolution. For β-CN, the spectral region of 195–260 nm was selected for secondary structure analysis, whereas for β-LG, the 190–260 nm range was used to minimize spectral interference caused by flavonoid absorption at shorter wavelengths while maintaining reliable secondary structure estimation. The estimated secondary structure contents of β-CN and β-LG are presented in Table 2.

For β-CN, CDNN analysis over 195–260 nm indicated that the control sample contained 42.3% total β-sheet structure, comprising 35.1% antiparallel and 7.2% parallel β-sheet, together with 22.3% α-helix, 20.1% β-turn, and 15.3% random coil.

Following catechin addition, the estimated α-helix content increased to 25.0% and 24.4% for β-CN–C1 and β-CN–C2, respectively, while the total β-sheet content decreased to 39.2% and 39.0%. These modest changes were consistent with a redistribution of the CDNN-estimated secondary-structure components but do not, by themselves, demonstrate the formation of a more ordered structure.

The naringin-containing samples exhibited smaller changes. Their estimated α-helix contents were 22.0% and 22.1%, while the total β-sheet contents were 40.8% and 41.4% for β-CN–NAR1 and β-CN–NAR2, respectively. Thus, naringin produced a comparatively limited effect on the estimated secondary-structure composition of β-CN under the tested conditions.

For β-LG, CDNN analysis over 190–260 nm indicated that the control sample contained 32.1% α-helix and 23.1% total β-sheet, including 15.4% antiparallel and 7.7% parallel β-sheet.

Catechin produced only minor changes in the estimated α-helix content, which was 32.5% and 32.4% for β-LG–C1 and β-LG–C2, respectively. In contrast, the antiparallel β-sheet content decreased slightly to 14.4% and 15.1%, while the total β-sheet content decreased to 22.2% and 22.8%.

Naringin produced a different trend. The antiparallel β-sheet content increased to 16.3% and 15.8% for β-LG–NAR1 and β-LG–NAR2, respectively, while the corresponding total β-sheet contents increased to 24.5% and 23.7%. The α-helix content decreased to 28.9% in β-LG–NAR1 and 31.1% in β-LG–NAR2.

These results indicate ligand-dependent variations in the CDNN-estimated secondary structure of β-LG. Catechin was associated with a slight decrease in the β-sheet fractions, whereas naringin produced a slight increase. Given the relatively small magnitude of these differences, the results do not support a general increase in β-sheet content following polyphenol addition.

### 3.4. Fluorescence Spectroscopy Analysis

**Figure 4:**
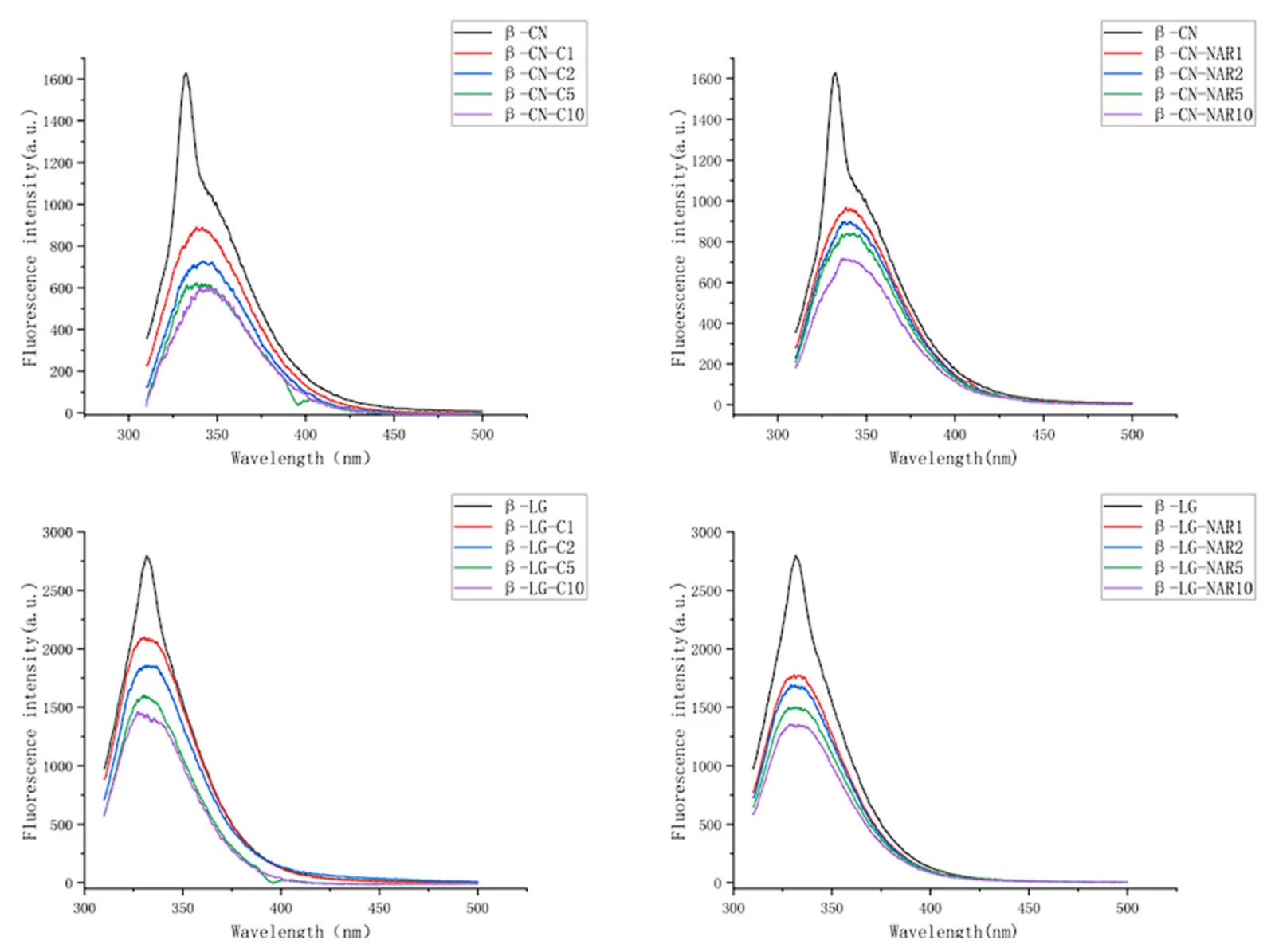
Intrinsic fluorescence spectra of two proteins incubated with flavonoids at different mass ratios All spectra shown are corrected for the intrinsic fluorescence of the corresponding flavonoid blank and for the inner filter effect. The final protein concentration was maintained at 1 μmol/L in all samples.

Fluorescence spectroscopy is widely used to investigate changes in the microenvironment polarity of aromatic amino acid residues (e.g., tyrosine (Tyr) and tryptophan (Trp)) and to infer alterations in protein tertiary structure. As shown in Figure 4, native β-CN and β-LG exhibited pronounced intrinsic fluorescence emission in the absence of polyphenols.

After the addition of catechin (C) and naringin (NAR), noticeable changes were observed in the fluorescence spectra of both β-CN and β-LG, characterized by a marked decrease in fluorescence intensity with increasing polyphenol concentration. In the β-CN system, at the highest polyphenol concentration (catechin 10 and naringin 10), the fluorescence intensity decreased from approximately 1620 a.u. to 600 a.u. and 700 a.u., respectively. Similarly, in the β-LG system, the fluorescence intensity decreased from approximately 2800 a.u. to 1450 a.u. and 1350 a.u., respectively. This pronounced fluorescence quenching suggests that catechin and naringin interact with both milk proteins, leading to changes in the microenvironment of aromatic residues (Trp and Tyr) and reduced exposure of intrinsic fluorophores. The extent of fluorescence quenching differed between β-CN and β-LG, suggesting that the two proteins exhibited different interaction behaviors toward flavonoids.

Furthermore, the addition of polyphenols induced a shift in the maximum emission wavelength, suggesting changes in the microenvironment of aromatic residues and possible alterations in protein conformation. In the β-CN–catechin system, a slight red shift was observed (from approximately 335 to 340 nm), indicating that tryptophan residues experienced a more polar microenvironment after binding. This behavior suggests that polyphenol–protein interactions may involve hydrophobic interactions with aromatic residues (Trp, Tyr, and Phe) and are accompanied by conformational rearrangements that modify the local environment of fluorophores. In contrast, in the β-LG system, a slight blue shift was observed (from approximately 332 to 330 nm), implying a relatively less polar microenvironment around tryptophan residues following polyphenol binding.

The observed fluorescence quenching and wavelength shifts suggest interactions between polyphenol molecules and both β-CN and β-LG. These changes indicate alterations in the microenvironment polarity of aromatic amino acid residues, accompanied by conformational rearrangements of the protein structure upon polyphenol binding. Collectively, these results provide molecular-level evidence for the interaction between milk proteins and polyphenols and contribute to a better understanding of their structure–function relationships.

### 3.5. Particle Size and Zeta Potential Analysis

**Figure 5:**
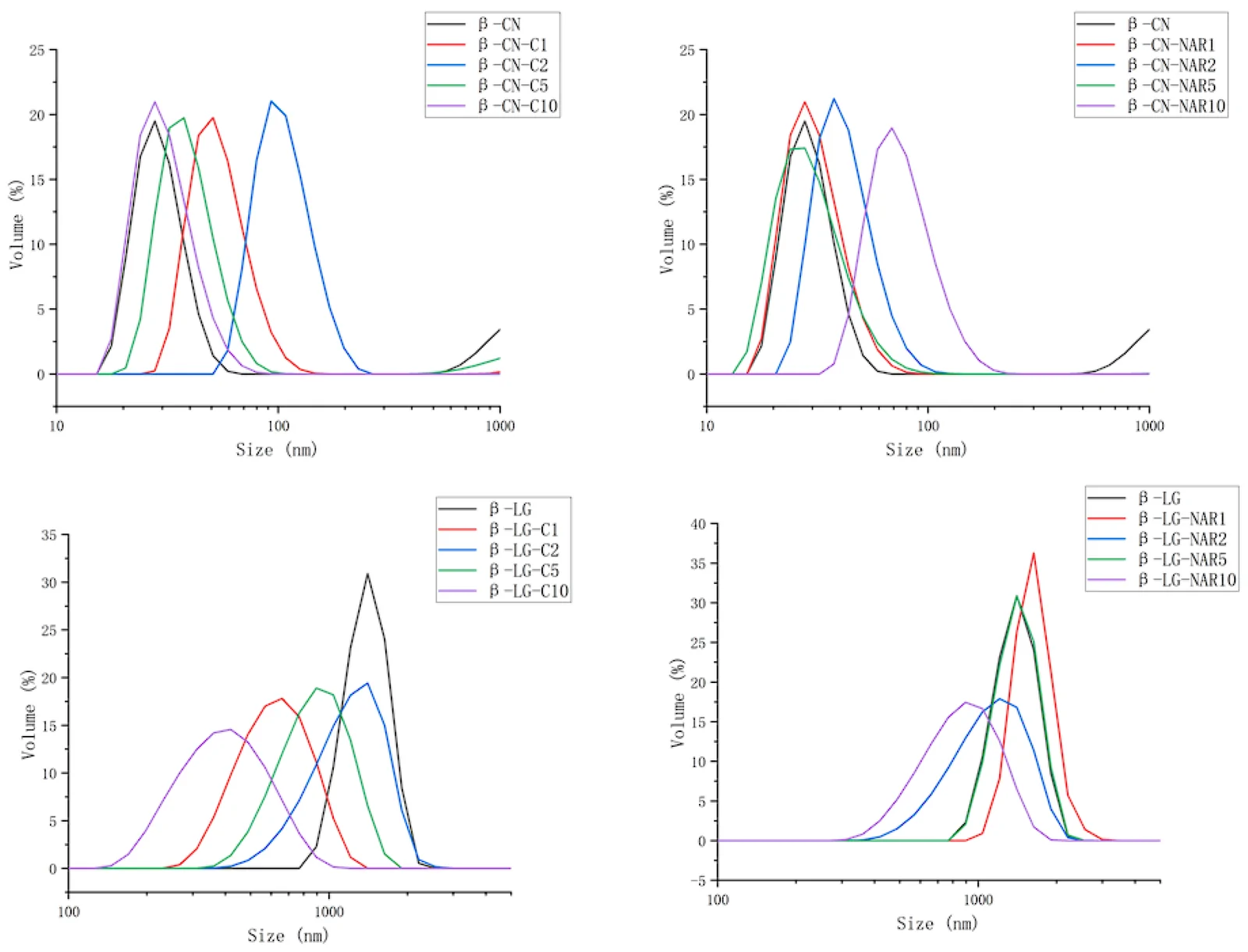
Volume-weighted particle-size distributions of milk proteins in the absence and presence of flavonoids at different ligand-to-protein molar ratios. β-CN with C; β-CN with NAR; β-LG with C; and β-LG with NAR. β-CN, β-casein; β-LG, β-lactoglobulin; C, catechin; NAR, naringin.

Particle size analysis revealed different concentration-dependent responses of β-casein (β-CN) and β-lactoglobulin (β-LG) following the addition of catechin or naringin (Figure 5).

Native β-CN exhibited a monomodal particle size distribution centered at approximately 30 nm. At the lower catechin ratio, the particle size increased to approximately 100 nm, which was consistent with the formation of larger protein-containing assemblies or transient aggregates. At higher catechin ratios, the particle size decreased to approximately 28–35 nm, indicating a concentration-dependent change in the association state of β-CN. However, the particle size data alone cannot determine whether this decrease resulted from dissociation of larger assemblies, redistribution of protein-containing particles, or other changes in the dispersion state.

At the higher examined ratios, naringin increased the particle size of β-CN to approximately 75 nm. This change was consistent with enhanced association or the formation of larger β-CN-containing assemblies. The different particle size responses produced by catechin and naringin may be related to their different molecular structures and interaction modes, although additional evidence is required to establish the underlying mechanism [25].

Native β-LG exhibited a broad particle size distribution centered at approximately 1500–2000 nm, indicating the presence of a population of relatively large particles consistent with protein aggregation. The addition of catechin or naringin reduced the particle size in a concentration-dependent manner. At the highest examined ratios, the particle size decreased to approximately 400 nm in the catechin-containing system and approximately 900 nm in the naringin-containing system. These changes were consistent with a redistribution or partial dissociation of the larger β-LG assemblies. Catechin therefore produced a greater decrease in the measured particle size than naringin under the tested conditions. Nevertheless, the particle size results alone do not provide a quantitative measure of binding affinity or interaction efficiency.

**Figure 6:**
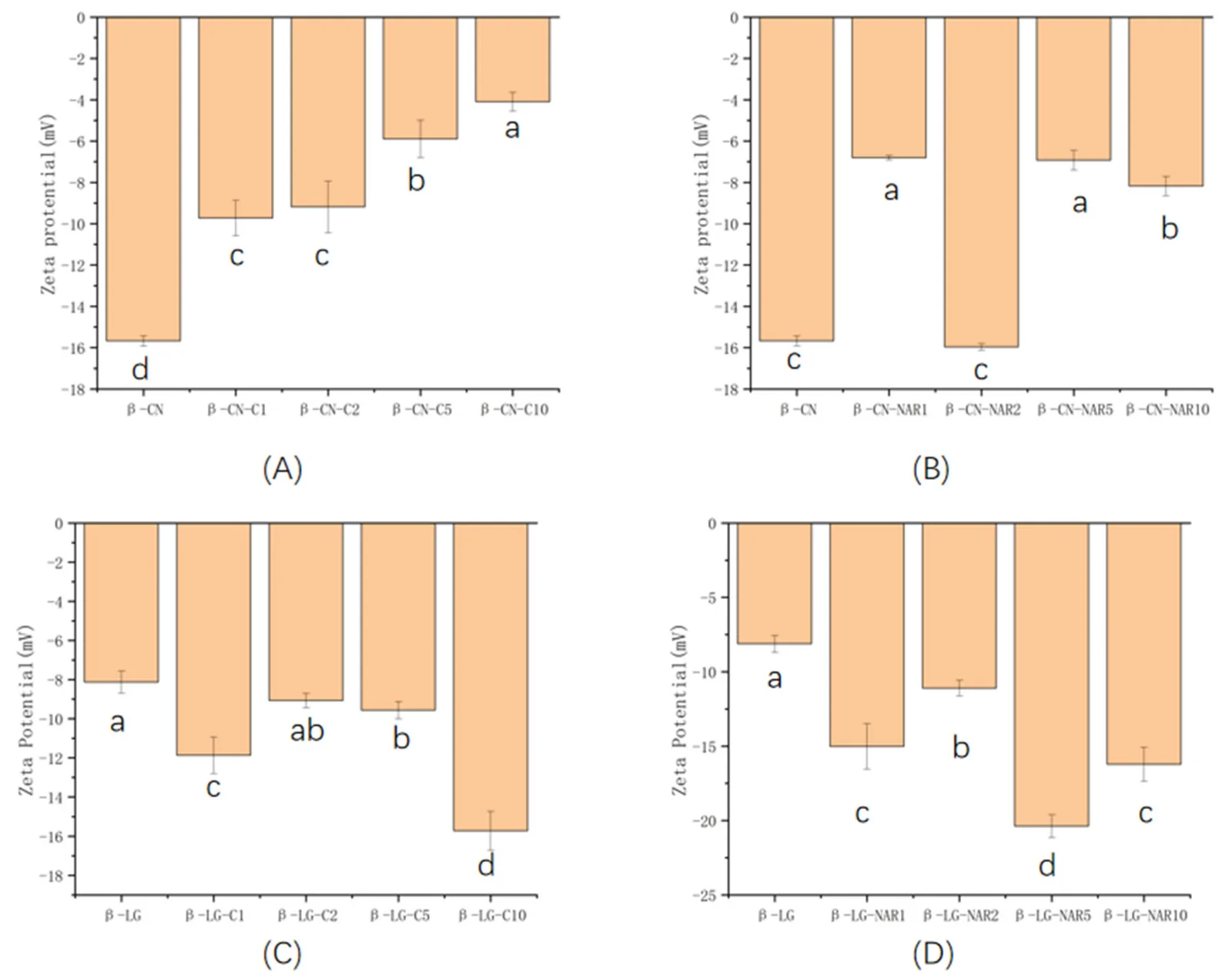
Effects of catechin and naringin on the zeta potentials of the protein–flavonoid systems: (**A**) β-CN–catechin, (**B**) β-CN–naringin, (**C**) β-LG–catechin, and (**D**) β-LG–naringin. Values are presented as mean ± SD (n = 3 independently prepared samples). Within each panel, statistical comparisons were performed using one-way ANOVA followed by Duncan’s multiple-range test. Different lowercase letters indicate significant differences among treatments (*p* < 0.05), whereas bars sharing at least one letter are not significantly different.

Zeta potential analysis further revealed changes in the electrokinetic properties of the aqueous protein–polyphenol systems (Figure 6). Native β-CN exhibited a zeta potential of −15.5 mV. Following catechin addition, the magnitude of the zeta potential gradually decreased, reaching −4.1 mV for β-CN–catechin 10. This change indicated a reduction in the net negative electrokinetic potential and may reflect charge screening, changes in the location of the slipping plane, or alterations in the interfacial composition. Naringin produced a similar but less uniform response.

Native β-LG exhibited a zeta potential of −8.1 mV. Following the addition of catechin or naringin, the zeta potential became more negative, reaching −15.8 mV for β-LG–catechin 10 and −20.4 mV for β-LG–naringin 5. The increase in the magnitude of the negative zeta potential indicated changes in the electrokinetic environment of the dispersed particles and may be associated with increased electrostatic repulsion. Together with the particle size results, these findings suggest that catechin and naringin altered the association and dispersion states of β-CN and β-LG in different ways. However, particle size and zeta potential measurements alone cannot establish the molecular mechanism of protein–polyphenol binding.

### 3.6. Molecular Docking Analysis

The top-ranked AutoDock Vina scores of β-CN–C, β-CN–NAR, β-LG–C, and β-LG–NAR were −5.2, −6.1, −6.1, and −7.4 kcal/mol, respectively. More negative values indicate more favorable predicted interactions under the applied scoring function. Thus, β-LG–NAR exhibited the most negative score, followed by β-CN–NAR and β-LG–C, whereas β-CN–C showed the least negative value [26].

**Figure 7:**
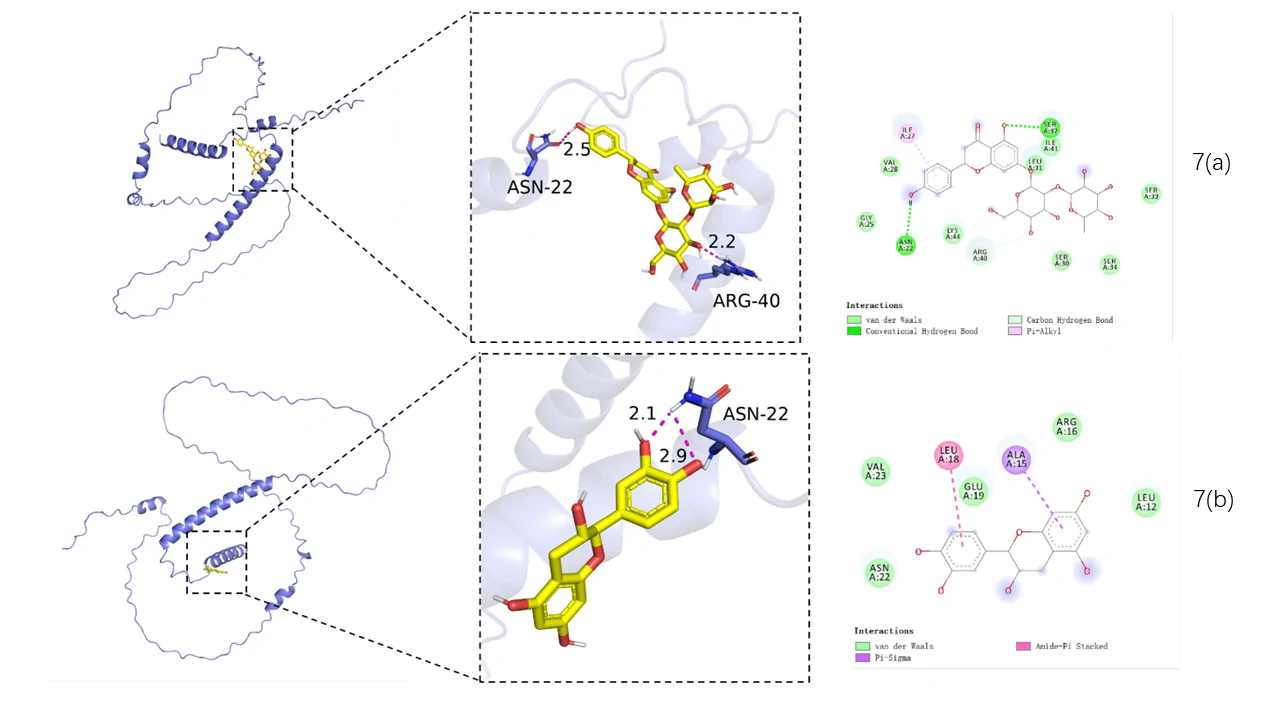

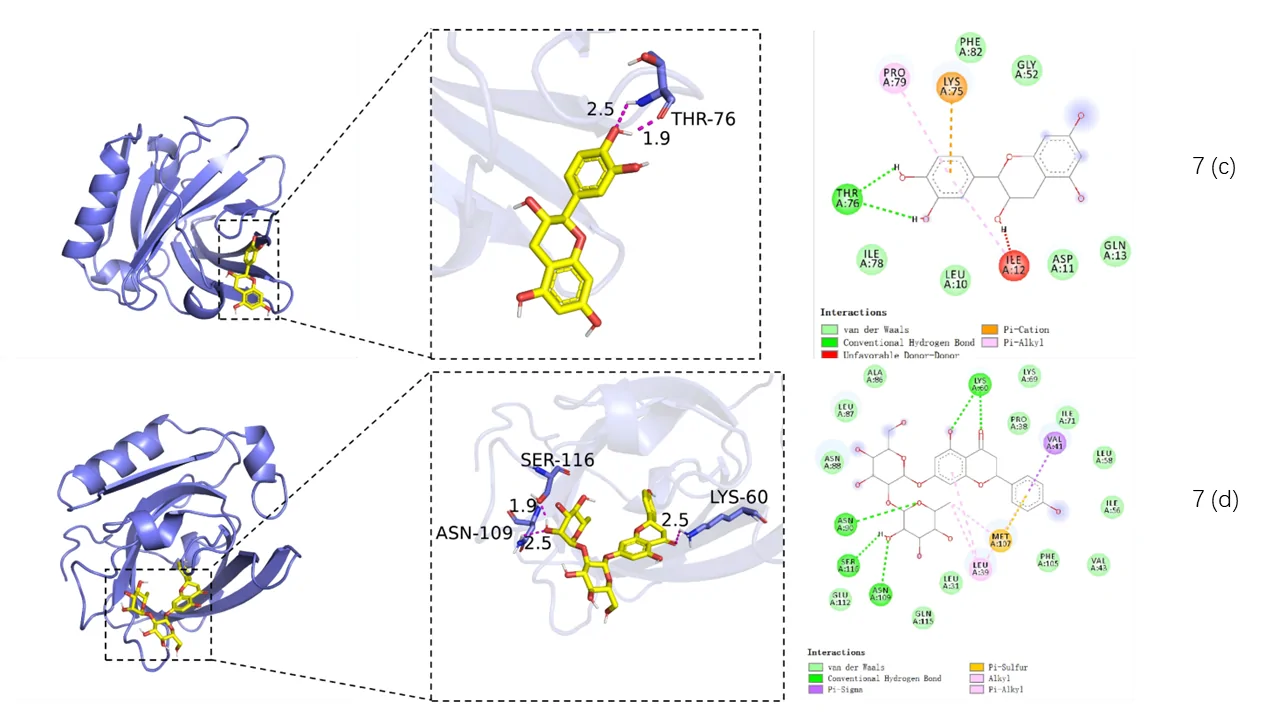
Predicted molecular docking poses and interaction diagrams of milk proteins with catechin and naringin. (**a**) β-CN–NAR, (**b**) β-CN–C, (**c**) β-LG–C, and (**d**) β-LG–NAR.

For β-CN–NAR (Figure 7a), conventional hydrogen bonds involved Asn22 and Ser37, together with a carbon–hydrogen contact with Arg40 and a π–alkyl interaction with Ile27. In β-CN–C (Figure 7b), close contacts with Asn22 and π interactions involving Ala15 and Leu18 were observed. For β-LG–C (Figure 7c), Thr76 formed two hydrogen bonds at approximately 1.9 and 2.5 Å, while Lys75 and Pro79 participated in π–cation and π–alkyl interactions, respectively [20]. β-LG–NAR (Figure 7d) formed hydrogen bonds with Lys60, Asn90, Asn109, and Ser116, together with π interactions involving Val41, Leu39, and Met107.

The β-LG–C score obtained in this study (−6.1 kcal/mol) was of the same general magnitude as the previously reported value of −6.824 kcal/mol [20], although direct comparison is limited by differences in docking protocols. Overall, the results indicated protein- and ligand-dependent differences in predicted interaction favorability. However, Vina scores are computational estimates rather than experimentally determined affinity parameters such as Kd or Ka, and the docking results based on the predicted β-CN structure should be interpreted cautiously [22].

### 3.7. Electronic Tongue Evaluation of Taste Characteristics and Bitterness Masking

The electronic tongue analysis revealed that the formation of protein–polyphenol complexes effectively reduced undesirable taste attributes in solution. In the free form, the naringin (NAR) and catechin (C) control groups exhibited pronounced bitterness intensities, reaching 2.50 and 1.78, respectively. In addition, free naringin showed an astringency response of 1.02. However, upon complexation with milk proteins, the bitterness and astringency of both polyphenols were markedly reduced, indicating that protein binding effectively masks their undesirable taste characteristics [27].

Specifically, the bitterness values of the β-CN–catechin and β-CN–naringin complexes decreased to 0.40 and 0.76, respectively. A similar trend was observed for β-LG, where the β-LG–catechin and β-LG–naringin complexes exhibited bitterness responses of 0.37 and 0.36, respectively. In addition, the astringency and aftertaste-bitterness (Aftertaste-B) indices of all protein–polyphenol complexes decreased to near-zero levels. These results indicate that complexation with milk proteins effectively reduces the bitterness and astringency of polyphenols in solution. This effect may be attributed to non-covalent interactions, including hydrogen bonding and hydrophobic interactions, which promote the formation of protein–polyphenol complexes and reduce the availability of free polyphenols in solution.

In addition to masking bitterness and astringency, the addition of proteins also modulated the overall taste profile of the mixed systems. The electronic tongue results showed that taste attributes such as sourness (all below −52.7) and richness (all below −2.5) remained at low response levels in all prepared solutions, indicating minimal contribution of these taste modalities. These results suggest that the protein–polyphenol mixed systems do not introduce additional undesirable taste attributes and maintain a relatively neutral taste profile.

Notably, all solution groups exhibited relatively high umami responses. The umami values of free naringin (NAR) and catechin (C) were 18.13 and 19.42, respectively. After complexation with milk proteins, the umami values of β-CN–catechin (20.18), β-CN–naringin (19.46), β-LG–catechin (20.32), and β-LG–naringin (20.16) were maintained at similar levels with slight variations. These results indicate that complexation with milk proteins does not adversely affect umami perception and may help preserve the intrinsic taste characteristics of the system. This behavior may be associated with interactions between milk proteins and polyphenols, which influence the distribution of taste-active compounds in solution [28].

The formation of protein–polyphenol macromolecular complexes effectively reduces bitterness while maintaining the overall taste profile of polyphenol-containing systems. These findings provide experimental evidence supporting the potential application of milk protein—polyphenol complexes in the development of functional beverage systems with improved sensory characteristics.

### 3.8. Integrated Structure–Electronic-Tongue Analysis

**Figure 8:**
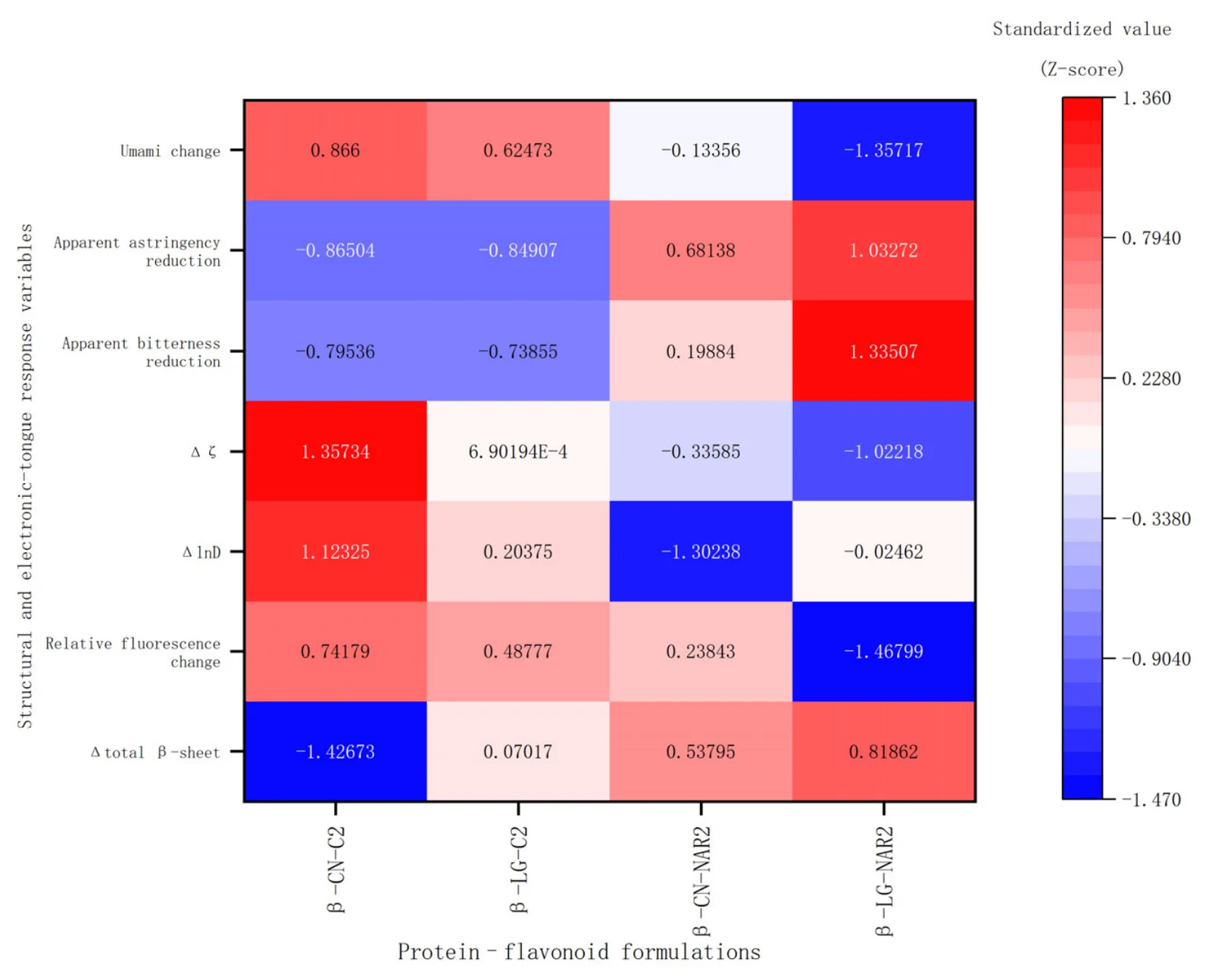
Integrated Z-score heatmap of structural changes and electronic-tongue responses in four protein–flavonoid formulations. Red and blue indicate values above and below the mean, respectively. The heatmap represents a descriptive comparison rather than a correlation analysis.

An integrated standardized heatmap was constructed to compare the structural changes and electronic-tongue responses of the four matched protein–flavonoid formulations (Figure 8). The two catechin-containing formulations showed higher-than-average umami changes but lower-than-average apparent reductions in bitterness and astringency. In contrast, the naringin-containing formulations exhibited comparatively greater apparent reductions in bitterness and astringency, accompanied by lower umami changes. Among the four formulations, β-LG–NAR2 showed the greatest relative apparent bitterness and astringency reductions and the lowest relative umami change.

Differences were also observed in the corresponding structural profiles. β-LG–NAR2 exhibited the highest standardized increase in total β-sheet content and the lowest relative fluorescence change, whereas β-CN–C2 showed the greatest positive changes in ΔlnD and Δζ and the largest relative decrease in total β-sheet content. Although some structural changes occurred concurrently with differences in the electronic-tongue responses, no single structural variable showed a uniform relationship with all three taste-related responses. Therefore, these matched patterns were interpreted as exploratory associations rather than statistically significant correlations.

## 4. Conclusions

The structural responses depended on both the protein and the flavonoid. For β-CN, catechin increased the estimated α-helix fraction from 22.3% to 24.4–25.0% and decreased the total β-sheet fraction from 42.3% to 39.0–39.2%, whereas naringin caused smaller changes. This difference may be related to the intrinsically disordered and flexible structure of β-CN, which contains surface-accessible interaction regions. The smaller catechin molecule may access these regions more readily, while the bulky glycosidic group of naringin may impose steric constraints.

By contrast, β-LG has a compact β-barrel structure containing an internal hydrophobic cavity. Flavonoid interactions may therefore remain localized within or near this cavity without causing extensive global rearrangement. Catechin slightly decreased the total β-sheet fraction of β-LG from 23.1% to 22.2–22.8%, whereas naringin increased it to 23.7–24.5%. These modest, opposite changes suggest ligand-dependent local rearrangements rather than a general effect of flavonoids on β-LG structure. Molecular docking provided qualitative support for possible hydrogen-bonding and hydrophobic interactions, but the predicted poses and scores should not be interpreted as experimentally determined binding sites or affinities.

Most importantly, the electronic-tongue analysis demonstrated the practical potential of milk proteins to attenuate bitterness- and astringency-related responses of catechin and naringin. Among the four matched formulations examined at the 1:2 protein-to-flavonoid molar ratio, β-LG–NAR2 showed the highest standardized apparent reductions in bitterness (Z = 1.34) and astringency (Z = 1.03), identifying it as a promising candidate for further formulation testing. These findings highlight the potential use of milk proteins as food-compatible taste-modulating components in flavonoid-containing dairy products and functional beverages. However, because the electronic tongue uses artificial sensor membranes and protein-only controls were not included, the observed responses cannot be directly equated with human taste perception. Validation in actual pandan-containing food matrices and by human sensory evaluation is therefore required.

These findings address the bitterness and astringency that may restrict consumer acceptance and diversification of flavonoid-rich pandan products. Technologically, milk proteins may serve as food-compatible taste-modulating components in pandan-based dairy products and functional beverages. Their application could support the development of value-added pandan products and broaden the utilization of pandan-derived ingredients. However, these technological and socioeconomic benefits remain prospective and require validation in actual pandan-containing formulations, quantitative affinity experiments, and human sensory studies.

## Figures and Tables

**Table 1:** Values are expressed as mean ± SD (n = 3). Secondary-structure contents were estimated using CDNN software and normalized to 100%. Different lowercase letters within the same column and protein group indicate significant differences according to one-way ANOVA followed by Duncan’s test (*p* < 0.05).

Samples	α-Helix (%)	β-Antiparallel (%)	β-Parallel (%)	β-Turn (%)	Random Coil (%)	Total (%)
β-CN“195-260nm”						
β-CN	22.3 ± 2.4 ^b^	35.1 ± 1.6 ^ab^	7.2 ± 0.96 ^a^	20.1 ± 0.68 ^a^	15.3 ± 0.38 ^a^	100 ± 0.1
β-CN-C1	25 ± 0.07 ^a^	32.3 ± 0.05 ^b^	6.9 ± 0.0 ^a^	21.1 ± 0.05 ^a^	14.5 ± 0.01 ^a^	99.8 ± 0.06
β-CN-C2	24.4 ± 0.05 ^a^	31.9 ± 0.01 ^b^	7.1 ± 0.0 ^a^	21 ± 0.0 ^a^	15.4 ± 0.05 ^a^	99.8 ± 0.05
β-CN-NAR1	22 ± 0.29 ^b^	33.3 ± 0.29 ^ab^	7.5 ± 0.03 ^a^	20.8 ± 0.04 ^a^	16.3 ± 0.02 ^a^	99.9 ± 0.0
β-CN-NAR2	22.1 ± 0.13 ^b^	34.1 ± 0.1 ^a^	7.3 ± 0.05 ^a^	20.9 ± 0.04 ^a^	15.5 ± 0.03 ^a^	99.9 ± 0.05
β-LG (190–260 nm)						
β-LG	33.8 ± 0.06 ^b^	12.2 ± 0.06 ^c^	8.2 ± 0.0 ^d^	18.1 ± 0.0 ^a^	27.5 ± 0.0 ^c^	99.8 ± 0.0
β-LG-C1	33.6 ± 0.12 ^b^	11.7 ± 0.01 ^d^	8.3 ± 0.0 ^c^	17.9 ± 0.01 ^c^	28.3 ± 0.14 ^b^	99.8 ± 0.06
β-LG-C2	34.8 ± 0.05 ^a^	11.5 ± 0.0 ^e^	8.1 ± 0.0 ^e^	18 ± 0.06 ^ab^	27.4 ± 0.4 ^d^	99.8 ± 0.0
β-LG-NAR1	29.6 ± 0.17 ^d^	13.2 ± 0.11 ^a^	8.9 ± 0.01 ^a^	18 ± 0.01 ^b^	30.4 ± 0.04 ^a^	100.1 ± 0.06
β-LG-NAR2	32.7 ± 0.16 ^c^	12.3 ± 0.1 ^b^	8.4 ± 0.06 ^b^	18.1 ± 0.0 ^a^	28.4 ± 0.12 ^b^	99.9 ± 0.1

**Table 2:** Electronic tongue taste profiles of two proteins after interaction with flavonoids.

Samples	Sourness	Bitterness	Astringency	Aftertaste-B	Aftertaste-A	Umami	Richness	Saltiness	Sweetness
β-CN-C	−61.21	0.40	−3.50	−0.82	0.36	20.18	−3.97	3.88	2.37
β-CN-NAR	−57.51	0.76	−2.15	−0.81	0.26	19.46	−3.25	1.29	2.22
β-LG-C	−61.88	0.38	−3.44	−0.83	0.35	20.32	−3.84	3.87	2.22
β-LG-NAR	−6	0.36	−3	−0.81	0.33	20.17	−3	3.86	2.31
C	−52.72	2.50	1.02	−0	0.21	18.13	−2	−4	2.93
NAR	−5	1.79	−0.86	−0.78	0.26	19.43	−3.17	−0	3.01

## Data Availability

The original contributions presented in this study are included in the article. Further inquiries can be directed to the corresponding authors.
